# Comparison of inequality in utilization of maternal healthcare services between Bangladesh and Pakistan: evidence from the demographic health survey 2017–2018

**DOI:** 10.1186/s12978-023-01595-y

**Published:** 2023-03-13

**Authors:** Farjana Misu, Khurshid Alam

**Affiliations:** 1 Murdoch Business School, Murdoch University, Perth, WA 6150 Australia; 2 Department of Statistics, Jagannath University, Dhaka-1100, Bangladesh

**Keywords:** Inequality, Maternal healthcare, Bangladesh, Pakistan

## Abstract

**Background:**

Inequality in maternal health has remained a challenge in many low-income countries, like Bangladesh and Pakistan. The study examines within-country and between-country inequality in utilization of maternal healthcare services for Bangladesh and Pakistan.

**Methods:**

The study used the latest Demographic Health Surveys (DHS, 2017–2018) datasets of Bangladesh and Pakistan for women aged 15–49 years who had given at least one live birth in three years preceding the survey. Equity strata were identified from the literature and conformed by binary logistic regressions. For ordered equity strata with more than two categories, the relative concentration index (RCI), absolute concentration index (ACI) and the slope index of inequality (SII) were calculated to measure inequalities in the utilization of four maternal healthcare services. For two-categories equity strata, rate ratio (RR), and rate difference (RD) were calculated. Concentration curves and equiplots were constructed to visually demonstrate inequality in maternal healthcare services.

**Results:**

In Bangladesh, there was greater inequality in skilled birth attendance (SBA) based on wealth (RCI: 0.424, ACI: 0.423, and SII: 0.612), women’s education (RCI: 0.380, ACI: 0.379 and SII: 0.591), husband’s education (RCI: 0.375, ACI: 0.373 and SII: 0.554) and birth order (RCI: − 0.242, ACI: − 0.241, and SII: -0.393). According to RCI, ACI, and SII, there was inequality in Pakistan for at least four ANC visits by the skilled provider based on wealth (RCI: 0.516, ACI: 0.516 and SII: 0.738), women’s education (RCI: 0.470, ACI: 0.470 and SII: 0.757), and husband’s education (RCI: 0.380, ACI: 0.379 and SII: 0.572). For Bangladesh, the RR (1.422) and RD (0.201) imply more significant urban–rural inequality in SBA. In Pakistan, urban–rural inequality was greater for at least four ANC visits by the skilled provider (RR: 1.650 and RD 0.279).

**Conclusion:**

Inequality in maternal healthcare is greater among the underprivileged group in Pakistan than in Bangladesh. In Bangladesh, the SBA is the most inequitable maternal healthcare, while for Pakistan it is at least four ANC visits by the skilled provider. Customized policies based on country context would be more effective in bridging the gap between the privileged and underprivileged groups.

**Supplementary Information:**

The online version contains supplementary material available at 10.1186/s12978-023-01595-y.

## Background

Inequality in maternal health is one of the key concerns for the low- and middle-income countries (LMICs) [1]. Health inequality must be addressed to reduce mortality and morbidity [2]. Most LMICs have substantially decreased maternal mortality ratio (MMR), even though these countries account for nearly all maternal mortality cases worldwide [3, 4]. Globally, the MMR dropped by about 38% from 2000 to 2017; nevertheless, LMICs experienced around 94% of global maternal deaths, while nearly 20% accounted for South Asia [5]. In South Asian developing nations, maternal mortality was caused by more than 40% of deliveries that took place outside of health facilities and 35% of births that did not occur under the observation of trained medical staff [6]. Inequality in the number of antenatal care (ANC) visits by the skilled provider, skilled birth attendance (SBA), and institutional delivery are associated with the high risks of maternal mortalities and morbidities [7, 8].

Therefore, maternal health remains a priority under the UN Sustainable Development Goals (SDGs) that emphasize equity. SDG 3 asks for ensuring healthy lives and fostering well-being for all, whereas SDG 10 calls for decreasing inequality within and between nations to promote inclusion and empowerment for all [9]. For maternal healthcare, governments must continue to gain momentum in reducing maternal mortality while focusing more on reducing inequities between population groups [10].

Among South Asian Countries, Bangladesh and Pakistan had high rates of maternal death (173 per 100,000 and 140 per 100,000, respectively) in 2017, which is a long way from the SDG target of fewer than 70 deaths per 100,000 live births by 2030 [11, 12]. Nearly half of women in Bangladesh get ANC from skilled health professionals, receive SBA and delivery in a health facility, which is far from meeting the goal in Bangladesh [13, 16]. Pakistan has more than 50% coverage of ANC and delivery care, but it is still challenging to reach the goal of reducing maternal mortality [15]. Many factors affected the utilization of maternal health services, and the widespread inequality in the utilization of health services was evident [17].

Social conditions, cultural beliefs, geographical and financial inaccessibility, and environmental conditions are the barriers to achieving equitable maternal healthcare services in LMICs [18]. Among South Asian countries, wealth-based, education-based, and region-based inequality are the barriers to achieving equality in maternal healthcare utilization [10, 19]. Healthcare utilization and health-related issues arise due to illiteracy, poor-rich gap, age, gender inequality, poor water quality and sanitation, unemployment, and geographical variation [20–23]. In Bangladesh, women with higher education, more media exposure, and a high family income are more likely to receive ANC services [24]. Additionally, it is demonstrated that in Bangladesh, religion, rural residency, household wealth, and education of both wife and husband are some of the crucial factors that lead to inequality in accessing to maternal healthcare services [25]. Women's low education, less autonomy in household decision-making, high birth order, and rural residency hinder the utilization of ANC in Pakistan [26]. For example, lack of knowledge regarding ANC, social barriers, financial constraints, non-acceptability of community midwives, high transport costs, and long-distance to health care facilities are the significant challenges to ANC and delivery care utilization in Pakistan [27].

**Fig. 1:**
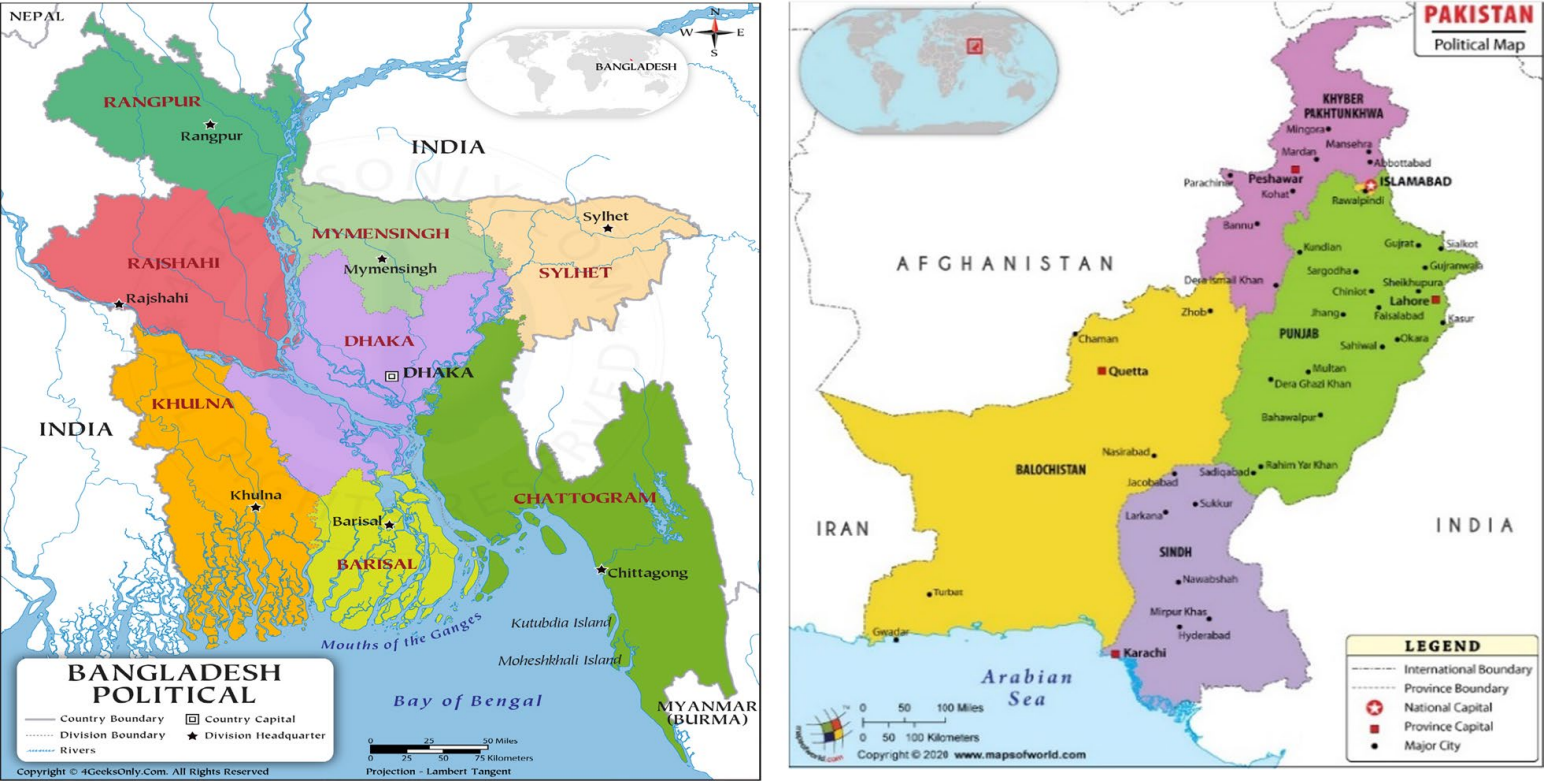
Map of Bangladesh and Pakistan. Source: https://www.burningcompass.com/countries/bangladesh/bangladesh-political-map-hd.html. Source: https://www.mapsofworld.com/pakistan/pakistan-political-map.html

Before 1971, Bangladesh and Pakistan shared single entity, known as East Pakistan and West Pakistan. The two countries shared common socio-political, religious, cultural, and economic backgrounds (Fig. 1). Political, regional, and socio-economic disparities result in an independent Bangladesh from Pakistan in 1971 [28]. Now after 50 years of separation, investigation of maternal healthcare utilization and comparing within and between the two countries would be an interesting exercise and ‘food for thought’ for the health policy makers in the region.

Several studies have measured inequality in key maternal healthcare indicators based on different equity strata in South Asia [12, 29, 30]. To the best of our knowledge, there is no such a study that attempts to identify within-country and between-country inequality in maternal healthcare services utilization for Bangladesh and Pakistan for the same period. The current study is an effort to help minimize this evidence gap. Hence, our study examines and compares inequality in four key maternal healthcare indicators using the latest and same period Demographic and Health Survey (DHS, 2017–2018) dataset and applying relative and absolute equality measures in common equity strata for Bangladesh and Pakistan.

## Methods

### Data

We analyzed nationally representative latest DHS datasets from Bangladesh and Pakistan collected during 2017–2018. The DHS collects detailed data on a wide range of maternal and child health issues in most LMICs once every five years. DHS is carried out in all regions of the country using a two-stage stratified sampling to choose families from the administrative regions of Bangladesh and Pakistan. The Bangladesh Demographic and Health Survey (BDHS) successfully interviewed 20,127 women aged 15–49 years out of a total of 20,376, resulting a response rate of 98.8% [31]. The Pakistan Demographic and Health Survey (PDHS) successfully interviewed 12,364 women aged 15–49 years out of a total 13,118, yielding a response rate of 94.3% [32]. In the current study, we restricted our sample to women who had given birth to at least one live birth in three years preceding the survey. When a woman had more than one live birth, we used data on the most recent live birth, resulting in a sample of women 4948 for Bangladesh and 5122 for Pakistan for the analysis.

### Outcome variables

The main outcome of interest of the study relates to the utilization of two important aspects of maternal healthcare: ANC and delivery care. In this study, we assessed ANC by two indicators: (i) single ANC visit in the first trimester by the skilled provider (Single ANC), and (ii) at least four ANC visits by skilled provider (Four + ANC). Similarly, we measured delivery care by two indicators: (i) SBA and (ii) facility-based delivery (FBD).

### Equity strata

Based on literature [33–37], we considered common equity strata (women’s age, women’s education, place of residence, women as household head, household size, employment status, wealth quintile, husband’s education, wanted last child, last live birth order and pregnancy termination history) to examine inequality in ANC and delivery care. Additional file 1: Table S1 provides the categorization and leveling of the outcome variables and equity strata.

### Statistical analysis

We carried out the empirical analyses in four different steps. Firstly, we undertook the descriptive analysis of the background characteristics of women who had given birth to at least one live birth in three years preceding the survey. We performed binary logistic regression models to identify and conform each equity stratum that impacted our selected maternal healthcare indicators. We calculated the utilization percentage of maternal healthcare indicators for each equity stratum. Finally, we employed relative and absolute inequality measures to identify inequality in maternal healthcare utilization based on equity strata, which significantly affect maternal healthcare utilization in Bangladesh or Pakistan. We used rate difference (RD) and rate ratio (RR) to measure absolute and relative inequality for ordered/non-ordered equity strata with two categories, such as place of residence, employment status, wanted last child, and history of pregnancy termination [38]. Since women’s age, women’s education, wealth quintile, husband’s education, and last live birth order are ordered equity strata with more than two categories, we employed concentration curve, relative concentration index (RCI) and absolute concentration index (ACI), and the slope index of inequality (SII) to examine inequality in maternal healthcare utilization [38]. Absolute inequality draws attention to the actual disparity in coverage between two extreme groups and the actions needed to bridge the gap. The degree of injustice between the privileged and the underprivileged is shown by relative inequality [39].

### Measuring inequality for ordered equity strata of more than two categories

The concentration curve is a graphical representation of inequality in maternal health care use that allows comparison of the degree of inequality across time periods or between nations. The concentration curve compares the cumulative proportion of maternal health care use indicators to the cumulative proportion of people ranked by various equity strata (such as wealth quintile and education). The line from the origin indicates perfect equality. The degree of inequality increases with the concentration curve's distance from the line of perfect equality. If the indicator of maternal healthcare use is concentrated among the privileged, the concentration curve is below the line of perfect equality; if it is concentrated among the underprivileged, it is above the line of perfect equality [20, 40].

Although the concentration curve is a useful tool to graphically demonstrate inequality, RCI, ACI and SII were employed to measure the magnitude of inequality in maternal health care use for common equity strata. Due to the concentration index's compliance with three criteria for a reliable socioeconomic inequality index [41], it is a widely used indicator of socioeconomic health inequities. The index should be responsive to the subpopulation group sizes, reflect health disparities resulting from socioeconomic features, and represent the entire population.

The RCI is based on the relative concentration curve, which is twice the area between the relative concentration curve and the perfect equality line. If the concentration curve is above (below) the line of equality, the RCI is negative (positive), indicating that the use of maternal healthcare services is concentrated among underprivileged (privileged) groups. The RCI ranges from − 1 to 1, with 0 denoting "perfect equality”. The RCI index can be calculated as follows [42]:

$$2{\sigma }_{r}^{2}\left(\frac{{h}_{i}}{\mu }\right)={\alpha }_{0}+ {\alpha }_{1}{r}_{i}+{\varepsilon }_{i},$$

where ${h}_{i}$ is the maternal healthcare variable of interest for $i\mathrm{th}$ women, μ is the mean of the maternal healthcare use variable for the whole sample, ${r}_{i}= \frac{i}{N}$, is the fractional rank of *i*th women in the distribution from the underprivileged woman ($i$ = 1) to the privileged woman ($i$ = N), and ${\sigma }_{r}^{2}$ is the variance of fractional rank. The ordinary least squares (OLS) estimate of ${\alpha }_{1}$ is used to determine the RCI.

Since our outcome variable of interest is binary, the minimum and maximum values of the RCI are not – 1 and + 1, thus, the RCI was normalized by multiplying the estimated index by $\frac{1}{1- \mu }$ [43, 44]. Absolute socioeconomic inequality in healthcare consumption can be calculated using the generalized concentration index. Since the generalized concentration index does not satisfy this condition, the *Erreygers* modified the generalized/ ACI (hereafter the $=RC \times 4\mu$) was used to calculate absolute inequality in maternal healthcare use [45]. The ACI ranges from − 1 to + 1, with zero suggesting perfect equality.

The SII is an absolute measure of inequality that considers all population subgroups. A weighted sample of the entire population is ordered from the disadvantaged subgroup (at rank 0) to the privileged subgroup to calculate SII (at rank 1). This rating is weighted to consider the population distribution within each category. The population of each subgroup is then considered in terms of its range and the midpoint of this range in the cumulative population distribution. Using a generalized linear model with a logit link, the health indicator of interest is then regressed on this midpoint value, and the projected values of the health indicator are generated for the two extremes (rank 1 and rank 0).

Therefore, the difference between the estimated values at rank 1 (${v}_{1}$) and rank 0 (${v}_{0}$) (covering the entire distribution) generates the SII value:

$$SII= {v}_{1}- {v}_{0}.$$

If there is no inequality, SII takes the value zero. Greater absolute values indicate higher levels of inequality. Positive values indicate a concentration of the indicator among the privileged, and negative values indicate a concentration of the indicator among the underprivileged [46, 47].

### Measuring inequality for equity strata with two categories

For equity strata with two categories (like place of residence, employment status, wanted last child, pregnancy termination history), RD and RR were calculated as following:

$$RD= {R}_{high}- {R}_{low},$$

$$RR= \frac{{R}_{high}}{{R}_{low}},$$

where, ${R}_{high}$ is the rate of healthcare use of women of the reference group (like urban/ not currently working/ wanted last child/ has pregnancy termination history), and ${R}_{low}$ is the rate of healthcare use of women of the non-referenced group. RR takes only positive values. The further the value of RR from 1, the higher the level of inequality. For RD, the larger the absolute value, the higher the level of inequality [46].

### Equiplots

To identify patterns of inequality, including linear, top, and bottom inequality, we constructed equiplots, which display the distance in healthcare coverage between different equity strata [39]. The equiplot is a data visualization tool that enables us to view all the indicators and their level of coverage simultaneously, providing a visual representation of absolute inequality [48].

All the statistical analyses were performed using STATA version 17.0.

## Results

Table 1 presents background characteristics of women aged 15–49 years who had given birth to at least one live birth in three years preceding the survey for Bangladesh and Pakistan. For Bangladesh, more than 75% of women were in the 20–34 years age category and majority lived in rural (73%) areas. The completion of higher secondary or above education rate for women and their husband was around 17% and 19%, respectively. Most women (around 87%) were not household heads, and 50% households had 1–5 members. Around two-thirds (63%) of women were not currently working. The last birth was wanted for the most women (79%), and 38% last live birth was the first child. Around 84% of women did not have a pregnancy termination history.

**Table 1:** Background characteristics of women of age 15–49 years of Bangladesh and Pakistan

Background characteristics	Bangladesh	Pakistan
Women’s age (years)		
15–19	18.01 (0.01)	4.76 (0.00)
20–34	76.21 (0.01)	78.73 (0.01)
35–49	5.78 (0.00)	16.51 (0.01)
Women’s education		
No formal schooling	6.33 (0.01)	47.91 (0.02)
Primary education not completed	17.38 (0.01)	5.68 (0.01)
Primary education completed	10.30 (0.01)	10.65 (0.01)
Junior school completed	43.58 (0.01)	11.68 (0.01)
Secondary school completed	5.25 (0.00)	10.51 (0.01)
Higher secondary or above	17.16 (0.01)	13.57 (0.01)
Place of residence		
Urban	26.78 (0.01)	32.91 (0.02)
Rural	73.22 (0.01)	67.09 (0.02)
Woman as household head		
Yes	13.20 (0.01)	11.08 (0.01)
No	86.80 (0.01)	88.92 (0.01)
Household size		
1–5 members	50.85 (0.01)	21.56 (0.01)
6 or more members	49.15 (0.01)	78.44 (0.01)
Employment status		
Currently employed	37.20 (0.01)	13.49 (0.01)
Not currently employed	62.80 (0.01)	86.51 (0.01)
Wealth quintile		
Poorest	20.63 (0.01)	21.60 (0.02)
Poorer	20.55 (0.01)	18.91 (0.01)
Middle	19.18 (0.01)	21.33 (0.01)
Richer	20.14 (0.01)	19.45 (0.01)
Richest	19.50 (0.01)	18.72 (0.01)
Husband’s education		
No formal schooling	13.70 (0.01)	29.18 (0.01)
Primary education not completed	19.28 (0.01)	4.99 (0.00)
Primary education completed	14.42 (0.01)	11.29 (0.01)
Junior school completed	28.43 (0.01)	16.84 (0.01)
Secondary school completed	5.66 (0.00)	18.06 (0.01)
Higher secondary or above	18.51 (0.01)	19.65 (0.01)
Wanted last child		
Yes	79.09 (0.01)	86.62 (0.01)
No	20.91 (0.01)	13.38 (0.01)
Last live birth order		
First	38.25 (0.01)	22.09 (0.01)
Second	32.77 (0.01)	21.86 (0.01)
Third	16.67 (0.01)	17.17 (0.01)
Fourth or higher	12.31 (0.01)	38.88 (0.01)
Pregnancy termination history		
Yes	16.40 (0.01)	29.58 (0.01)
No	83.60 (0.01)	70.42 (0.01)

Background characteristics of women are given in percentage and standard error are in parenthesis. Standard error is too small but not zero in some cases. Due to rounding off the values, the total percentage may not be a hundred

For Pakistan, the most women (79%) were in the 20–34 years age category and live in rural (67%) areas. The completion of higher secondary or above education rate for women and their husband was around 14% and 20%, respectively. Most women (around 89%) were not household heads, and around 78% households had six or more members. Around 87% of women were not currently working. Most women wanted their last child (87%), and around 39% of the last live birth were fourth or higher order. Around 70% of women did not have a pregnancy termination history.

The binary logistic regression models assessed the impact of each equity stratum on each outcome variable for Bangladesh and Pakistan (Additional file 1: Table S2). Women’s education, place of residence, employment status, wealth quintile, husband’s education, and last live birth order had a significant (p < 0.05) impact on maternal healthcare indicators for Bangladesh and Pakistan. In Bangladesh, wanted last child had a significant (p < 0.05) impact on maternal healthcare indicators, but this had no impact on Pakistan. Pregnancy termination history impacted a few maternal healthcare indicators for Bangladesh and Pakistan.

Table 2 exhibits the coverage of four maternal healthcare utilization indicators by common equity strata for Bangladesh and Pakistan, where we observed considerable differences between these two countries. In Pakistan, the coverage of SBA and FBD was about 73% and 70%, respectively, while in Bangladesh, it was around 53% and 50%, respectively. The rate of single ANC visits by a skilled provider was 37% in Bangladesh and 55% in Pakistan. For SBA and FBD, the utilization was 52–53% for higher secondary and above education than for no formal education in Bangladesh. In Pakistan, it was 36–38% for higher secondary and above education than for no education for SBA and FBD.

**Table 2:** Coverage of maternal healthcare by common equity strata for Bangladesh and Pakistan

Equity strata	Bangladesh					Pakistan				
	Sample size (n)	Single ANC visit (1st trimester) by skilled provider	At least four ANC visits by skilled provider	Skilled birth attendance	Facility-based delivery	Sample size (n)	Single ANC visit (1st trimester) by skilled provider	At least four ANC visits by skilled provider	Skilled birth attendance	Facility-based delivery
National	4948	37.15 (0.01)	47.07 (0.01)	52.98 (0.01)	49.70 (0.01)	5122	54.83 (0.01)	52.17 (0.02)	73.37 (0.01)	70.35 (0.02)
Women’s age										
15–19	864	35.55 (0.02)	47.49 (0.02)	54.54 (0.02)	50.73 (0.02)	279	54.81 (0.04)	45.43 (0.04)	70.87 (0.04)	67.74 (0.04)
20–34	3782	38.04 (0.01)	47.59 (0.01)	53.01 (0.01)	49.98 (0.01)	3948	56.57 (0.01)	54.01 (0.02)	74.83 (0.02)	72.01 (0.02)
35–49	302	30.47 (0.03)	38.98 (0.03)	47.75 (0.03)	42.80 (0.03)	895	46.54 (0.02)	45.35 (0.03)	67.13 (0.03)	63.20 (0.03)
Women’s education										
No formal schooling	307	19.97 (0.02)	19.78 (0.02)	29.06 (0.03)	26.50 (0.03)	2752	38.36 (0.01)	31.71 (0.02)	59.74 (0.02)	56.00 (0.02)
Primary education not completed	853	24.16 (0.02)	32.55 (0.02)	32.01 (0.02)	29.43 (0.02)	229	55.76 (0.04)	52.83 (0.04)	72.72 (0.03)	69.66 (0.04)
Primary education completed	524	30.00 (0.02)	36.20 (0.03)	37.82 (0.03)	35.00 (0.03)	445	56.24 (0.03)	54.76 (0.03)	79.53 (0.02)	75.38 (0.02)
Junior school completed	2106	36.29 (0.01)	49.37 (0.01)	54.48 (0.02)	50.64 (0.01)	504	70.36 (0.03)	69.82 (0.03)	82.65 (0.02)	80.68 (0.02)
Secondary school completed	262	44.73 (0.03)	58.39 (0.03)	74.74 (0.03)	71.63 (0.03)	490	76.58 (0.03)	77.95 (0.03)	90.34 (0.02)	88.51 (0.02)
Higher secondary or above	896	60.80 (0.02)	69.08 (0.02)	81.70 (0.02)	78.50 (0.02)	702	81.32 (0.02)	86.98 (0.02)	95.79 (0.01)	94.42 (0.01)
Place of residence										
Urban	1697	47.33 (0.02)	58.71 (0.02)	67.69 (0.02)	62.82 (0.02)	2353	68.84 (0.02)	70.93 (0.02)	86.35 (0.02)	83.64 (0.01)
Rural	3251	33.43 (0.01)	42.82 (0.01)	47.61 (0.01)	44.90 (0.01)	2769	47.96 (0.02)	42.98 (0.02)	67.00 (0.02)	63.83 (0.02)
Employment status										
Currently employed	1853	31.53 (0.01)	45.42 (0.02)	44.26 (0.02)	40.79 (0.02)	580	49.18 (0.03)	44.90 (0.04)	68.40 (0.03)	66.03 (0.03)
Not currently employed	3095	40.48 (0.01)	48.05 (0.01)	58.15 (0.01)	54.97 (0.01)	4541	55.71 (0.01)	53.31 (0.02)	74.14 (0.01)	71.03 (0.02)
Wealth quintile										
Poorest	1066	22.10 (0.01)	30.95 (0.02)	27.93 (0.02)	26.35 (0.02)	1164	27.38 (0.02)	22.51 (0.02)	51.52 (0.03)	47.76 (0.03)
Poorer	1007	30.03 (0.02)	36.40 (0.02)	40.35 (0.02)	37.26 (0.02)	1029	41.68 (0.02)	35.43 (0.02)	59.87 (0.03)	56.46 (0.03)
Middle	892	35.38 (0.02)	45.73 (0.02)	52.86 (0.02)	49.07 (0.02)	972	57.78 (0.02)	50.57 (0.03)	77.13 (0.02)	73.41 (0.02)
Richer	972	38.36 (0.02)	51.78 (0.02)	62.94 (0.02)	59.25 (0.02)	940	69.16 (0.02)	70.87 (0.02)	86.62 (0.02)	83.91 (0.02)
Richest	1011	61.06 (0.02)	71.84 (0.02)	82.64 (0.01)	78.26 (0.01)	1017	81.56 (0.02)	85.73 (0.03)	94.17 (0.01)	92.88 (0.01)
Husband’s education										
No formal schooling	672	22.32 (0.02)	29.88 (0.02)	31.73 (0.02)	28.91 (0.02)	1505	36.69 (0.02)	30.73 (0.02)	59.17 (0.02)	55.06 (0.02)
Primary education not completed	927	27.18 (0.02)	34.57 (0.02)	37.92 (0.02)	35.22 (0.02)	241	48.25 (0.04)	41.15 (0.04)	65.77 (0.05)	62.66 (0.04)
Primary education completed	707	28.51 (0.02)	40.69 (0.02)	44.81 (0.02)	40.75 (0.02)	488	51.35 (0.03)	45.73 (0.03)	68.05 (0.03)	64.78 (0.03)
Junior school completed	1352	37.83 (0.02)	49.38 (0.02)	56.31 (0.02)	53.01 (0.02)	789	60.38 (0.02)	57.06 (0.03)	74.91 (0.03)	71.26 (0.03)
Secondary school completed	267	49.07 (0.04)	57.06 (0.03)	70.50 (0.03)	65.55 (0.03)	901	63.53 (0.02)	63.78 (0.02)	82.46 (0.02)	80.63 (0.02)
Higher secondary or above	948	60.79 (0.02)	71.94 (0.02)	81.19 (0.02)	77.98 (0.02)	1146	73.12 (0.02)	75.90 (0.02)	89.69 (0.01)	87.87 (0.01)
Wanted last child										
Yes	3902	38.98 (0.01)	49.33 (0.01)	55.32 (0.01)	52.17 (0.01)	4549	54.78 (0.01)	52.11 (0.02)	73.08 (0.02)	70.23 (0.02)
No	1046	30.23 (0.02)	38.52 (0.02)	44.15 (0.02)	40.36 (0.02)	573	55.19 (0.03)	52.57 (0.03)	75.24 (0.02)	71.11 (0.02)
Last live birth order										
First	1893	42.68 (0.02)	53.79 (0.02)	64.53 (0.02)	60.81 (0.02)	1092	70.44 (0.02)	64.72 (0.02)	83.39 (0.02)	81.95 (0.02)
Second	1614	38.14 (0.01)	48.21 (0.02)	52.29 (0.02)	49.56 (0.02)	1121	60.97 (0.02)	57.47 (0.02)	77.78 (0.02)	74.44 (0.02)
Third	840	33.03 (0.02)	44.70 (0.02)	44.79 (0.02)	41.59 (0.02)	907	53.80 (0.03)	52.59 (0.03)	75.10 (0.02)	72.40 (0.02)
Fourth or higher	601	22.90 (0.02)	26.40 (0.02)	30.04 (0.02)	26.51 (0.02)	2002	42.97 (0.02)	41.89 (0.02)	64.43 (0.02)	60.56 (0.02)
Pregnancy termination history										
Yes	831	39.05 (0.02)	51.65 (0.02)	56.80 (0.02)	54.15 (0.02)	1423	57.95 (0.02)	54.71 (0.02)	71.90 (0.02)	69.58 (0.02)
No	4117	36.78 (0.01)	46.17 (0.01)	52.23 (0.01)	48.82 (0.01)	3699	53.52 (0.01)	51.11 (0.02)	73.99 (0.01)	70.67 (0.02)

Coverage of maternal healthcare indicators is given in percentage and standard error is in parenthesis

The urban–rural gap in a single ANC and at least four ANC from qualified providers differs by 21–28% in Pakistan, and this gap was 14–16% in Bangladesh. The disparity by employment status for SBA and FBD was 14% higher in Bangladesh, and 5–6% higher in Pakistan. Within the country, the rich-poor disparity in SBA and FBD was 52–55% higher for the richest than the poorest in Bangladesh, and in Pakistan, it was 42–45% higher. On the contrary, the rich-poor disparity in a single ANC and at least four ANC from qualified providers was 55–63% higher in Pakistan, and in Bangladesh, it was 39–41% higher. In Bangladesh, for the first-order child, maternal healthcare utilization was around 65% in SBA and 61% in FBD, while in Pakistan, it was 83% and 82%, respectively. The utilization of maternal healthcare among women with a pregnancy termination history was about 57% in SBA and 54% in FBD for Bangladesh, while for Pakistan, it was about 72% in SBA and 70% in FBD.

**Fig. 2:**
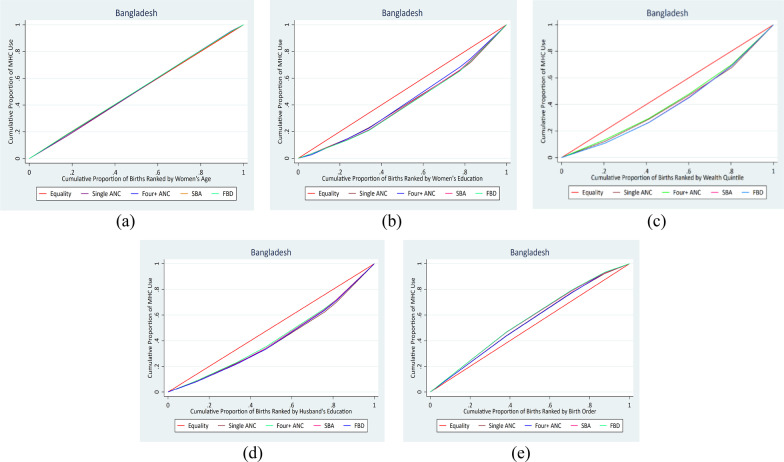
Concentration curves of equity strata for maternal healthcare utilization in Bangladesh

**Fig. 3:**
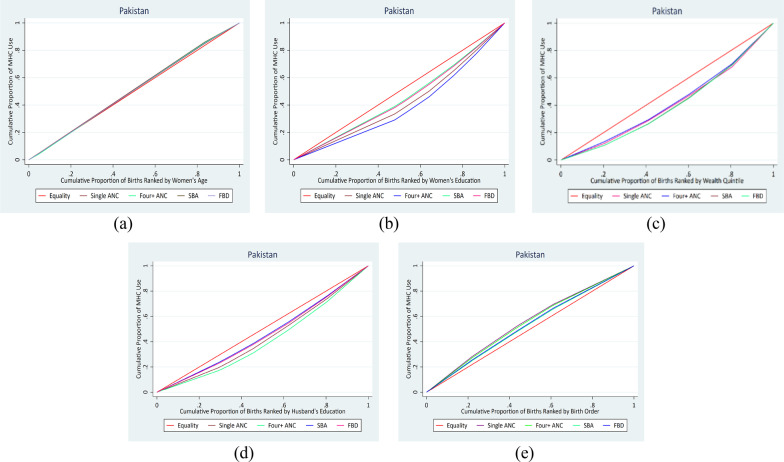
Concentration curves of equity strata for maternal healthcare utilization in Pakistan

Figures 2 and 3 represent concentration curves for all four maternal healthcare use indicators by women’s age, education, wealth, husband’s education, and last live birth order for Bangladesh and Pakistan. The concentration curves close to the line of equality in Figs. 2a and 3a show no inequality in the utilization of maternal healthcare based on women’s age in Bangladesh and Pakistan. The concentration curves of all the maternal healthcare utilization indicators under the line of equality imply that inequality was disproportionately concentrated in the women who completed higher education (Figs. 2b and 3b), women in the richest wealth quintile (Figs. 2c and 3c), and women whose husbands completed higher education (Figs. 2d and 3d) for both Bangladesh and Pakistan. The concentration curves of all the maternal healthcare utilization indicators above the line of equality indicated that inequality was concentrated in the first-order child (Figs. 2e and 3e) of women in Bangladesh and Pakistan.

Table 3 shows the relative and absolute measure of inequality for maternal healthcare utilization indicators based on women’s age, education, wealth, husband’s education, and last live birth order for Bangladesh and Pakistan. Positive values of RCI, ACI and SII indicated that the respective maternal healthcare utilization indicators were more concentrated among women in the richest wealth quintile who completed higher education and whose husbands had higher education in Bangladesh and Pakistan. Negative values of RCI, ACI and SII indicated that the maternal healthcare utilization indicators were more concentrated among women of the lowest age group and for the first-order child in both countries.

**Table 3:** Relative and absolute inequality index of maternal healthcare use by common equity strata

Inequality measures	Bangladesh				Pakistan			
	Single ANC visit (1st trimester) by skilled provider	At least four ANC visits by skilled provider	Skilled birth attendance	Facility-based delivery	Single ANC visit (1st trimester) by skilled provider	At least four ANC visits by skilled provider	Skilled birth attendance	Facility-based delivery
Women’s age								
Relative Concentration Index (RCI)	− 0.002 (0.01)	− 0.018 (0.01)	− 0.021 (0.01)	− 0.020 (0.01)	− 0.053 (0.01)	− 0.032 (0.02)	− 0.045 (0.02)	− 0.049 (0.02)
Absolute Concentration Index (ACI)	− 0.002 (0.01)	− 0.018 (0.01)	− 0.020 (0.01)	− 0.020 (0.01)	− 0.052 (0.01)	− 0.032 (0.02)	− 0.035 (0.02)	− 0.041 (0.02)
Slope Index of inequality (SII)	− 0.0004 (0.04)	− 0.044 (0.04)	− 0.052 (0.04)	− 0.050 (0.04)	− 0.148 (0.04)	− 0.080 (0.05)	− 0.095 (0.05)	− 0.110 (0.05)
Women’s education								
Relative Concentration Index (RCI)	0.277 (0.02)	0.299 (0.02)	0.380 (0.02)	0.373 (0.02)	0.380 (0.02)	0.470 (0.02)	0.394 (0.03)	0.392 (0.03)
Absolute Concentration Index (ACI)	0.259 (0.02)	0.298 (0.02)	0.379 (0.02)	0.373 (0.02)	0.376 (0.02)	0.470 (0.02)	0.308 (0.02)	0.327 (0.02)
Slope Index of inequality (SII)	0.416 (0.03)	0.476 (0.03)	0.591 (0.02)	0.582 (0.02)	0.644 (0.03)	0.757 (0.02)	0.611 (0.04)	0.627 (0.04)
Wealth status								
Relative Concentration Index (RCI)	0.294 (0.02)	0.311 (0.02)	0.424 (0.02)	0.403 (0.02)	0.439 (0.02)	0.516 (0.02)	0.459 (0.03)	0.451 (0.03)
Absolute Concentration Index (ACI)	0.275 (0.02)	0.310 (0.02)	0.423 (0.02)	0.403 (0.02)	0.435 (0.02)	0.516 (0.02)	0.358 (0.03)	0.376 (0.03)
Slope Index of inequality (SII)	0.412 (0.03)	0.461 (0.03)	0.612 (0.02)	0.586 (0.02)	0.645 (0.02)	0.738 (0.02)	0.569 (0.04)	0.587 (0.04)
Husband’s education								
Relative Concentration Index (RCI)	0.300 (0.02)	0.310 (0.02)	0.375 (0.02)	0.368 (0.02)	0.306 (0.02)	0.380 (0.02)	0.332 (0.03)	0.335 (0.02)
Absolute Concentration Index (ACI)	0.281 (0.02)	0.309 (0.02)	0.373 (0.02)	0.368 (0.02)	0.303 (0.02)	0.379 (0.02)	0.260 (0.02)	0.280 (0.02)
Slope Index of inequality (SII)	0.426 (0.03)	0.466 (0.03)	0.554 (0.02)	0.546 (0.02)	0.468 (0.03)	0.572 (0.03)	0.424 (0.03)	0.451 (0.03)
Last birth order								
Relative Concentration Index (RCI)	− 0.138 (0.02)	− 0.161 (0.02)	− 0.242 (0.02)	− 0.237 (0.02)	− 0.241 (0.02)	− 0.200 (0.02)	− 0.213 (0.02)	− 0.221 (0.02)
Absolute Concentration Index (ACI)	− 0.129 (0.02)	− 0.161 (0.02)	− 0.241 (0.02)	− 0.237 (0.02)	− 0.239 (0.02)	− 0.200 (0.02)	− 0.166 (0.02)	− 0.184 (0.02)
Slope Index of inequality (SII)	− 0.217 (0.03)	− 0.267 (0.03)	− 0.393 (0.03)	− 0.388 (0.03)	− 0.369 (0.03)	− 0.312 (0.03)	− 0.261 (0.03)	− 0.288 (0.03)

Maternal age is categorized as ‘15–19’, ‘20–34’ and ‘35–49’. Women’s education and husband’s education are categorized as no formal schooling, primary education not completed, primary education completed, junior school completed, secondary school completed and higher secondary or above. Birth order is categorized as ‘First’, ‘Second’ ‘Third’ and ‘Forth or higher’. Standard error is in parenthesis

In Bangladesh, the values of RCI, ACI and SII (Table 3) suggest that the level of inequality was higher in SBA and lowest in single ANC visit (1st trimester) by the skilled provider based on wealth (SBA- RCI: 0.424, ACI: 0.423, SII: 0.612 and single ANC- RCI: 0.294, ACI: 0.275, SII: 0.412), women’s education (SBA- RCI: 0.380, ACI: 0.379, SII: 0.591 and single ANC- RCI: 0.277, ACI: 0.259, SII: 0.416), and husband’s education (SBA- RCI: 0.375,ACI: 0.373, SII: 0.554 and single ANC- RCI: 0.300, ACI: 0.281, SII: 0.426). In Pakistan, according to the values of RCI, ACI and SII, the level of inequality was higher for at least four ANC visits by the skilled provider among all the four maternal healthcare indicators based on wealth (RCI: 0.516, ACI: 0.516 and SII: 0.738), women’s education (RCI: 0.470, ACI: 0.470 and SII: 0.757), and husband’s education (RCI: 0.380, ACI: 0.379 and SII: 0.572). For wealth status, women’s education, and husband’s education, the values of ACI (0.358, 0.308, and 0.260, respectively) and SII (0.569, 0.611, and 0.424, respectively) revealed a lower level of inequality in SBA in Pakistan.

In Table 3, the values of the RCI, ACI, and SII close to zero indicated a shallow inequality in maternal healthcare utilization indicators according to women’s age for Bangladesh and Pakistan. According to birth order, inequality was highest in SBA (RCI: − 0.242, ACI: − 0.241, and SII: − 0.393) and lowest in single ANC visits (1st trimester) by the skilled provider (RCI: − 0.138, ACI: − 0.129 and SII: − 0.217) among all maternal healthcare use indicators in Bangladesh. In Pakistan, inequality was highest in single ANC visit (1st trimester) by the skilled provider for birth order among all maternal healthcare use indicators.

Table 4 shows relative (RR) and absolute (RD) inequality measures for the maternal healthcare utilization indicators by common equity strata for Bangladesh and Pakistan. Positive values of RR and RD indicate that the maternal healthcare indicators were more concentrated among women in urban areas, who were not currently working, wanted the last child and had a pregnancy termination history. For Bangladesh, the RR (1.422) and RD (0.201) indicates a more significant urban–rural inequality in SBA among all four maternal healthcare indicators. For Pakistan, the level of urban–rural inequality was greater for at least four ANC visits by the skilled provider and lower in SBA according to the RR (1.650 and 1.289, respectively) and RD (0.279 and 0.193, respectively). Women’s employment-related inequality was highest for FBD in Bangladesh (RR: 1.348, RD: 0.142), while in Pakistan, it was highest for at least four ANC visits by the skilled provider (RR: 1.187, RD:0.084). The RR and RD values indicated the lowest level of wanted last child-related, and pregnancy termination-related inequality for Bangladesh and Pakistan.

**Table 4:** Relative and absolute inequality measure of maternal healthcare use by common equity strata

Inequality measures	Bangladesh				Pakistan			
	Single ANC visit (1st trimester) by skilled provider	At least four ANC visits by skilled provider	Skilled birth attendance	Facility-based delivery	Single ANC visit (1st trimester) by skilled provider	At least four ANC visits by skilled provider	Skilled birth attendance	Facility-based delivery
Urban–rural								
Rate ratio (ref: urban)	1.416	1.371	1.422	1.399	1.435	1.650	1.289	1.310
Rate difference (ref: urban)	0.139	0.159	0.201	0.179	0.209	0.279	0.193	0.198
Employment status								
Rate ratio (ref: not currently employed)	1.284	1.058	1.314	1.348	1.133	1.187	1.084	1.076
Rate difference (ref: not currently employed)	0.090	0.026	0.139	0.142	0.065	0.084	0.057	0.050
Wanted last child								
Rate ratio (ref: yes)	1.289	1.281	1.253	1.293	0.992	0.991	0.971	0.988
Rate difference (ref: yes)	0.087	0.108	0.112	0.118	− 0.004	− 0.005	− 0.022	− 0.009
Pregnancy termination history								
Rate ratio (ref: yes)	1.062	1.119	1.087	1.109	1.083	1.071	0.972	0.985
Rate difference (ref: yes)	0.023	0.055	0.046	0.053	0.044	0.036	− 0.021	− 0.011

Employment is categorized as currently working (having worked in the past 7 days, including women who did not work in the past 7 days but who are regularly employed and were absent from work for leave, illness, vacation, or any other such reason) and not currently working

**Fig. 4:**
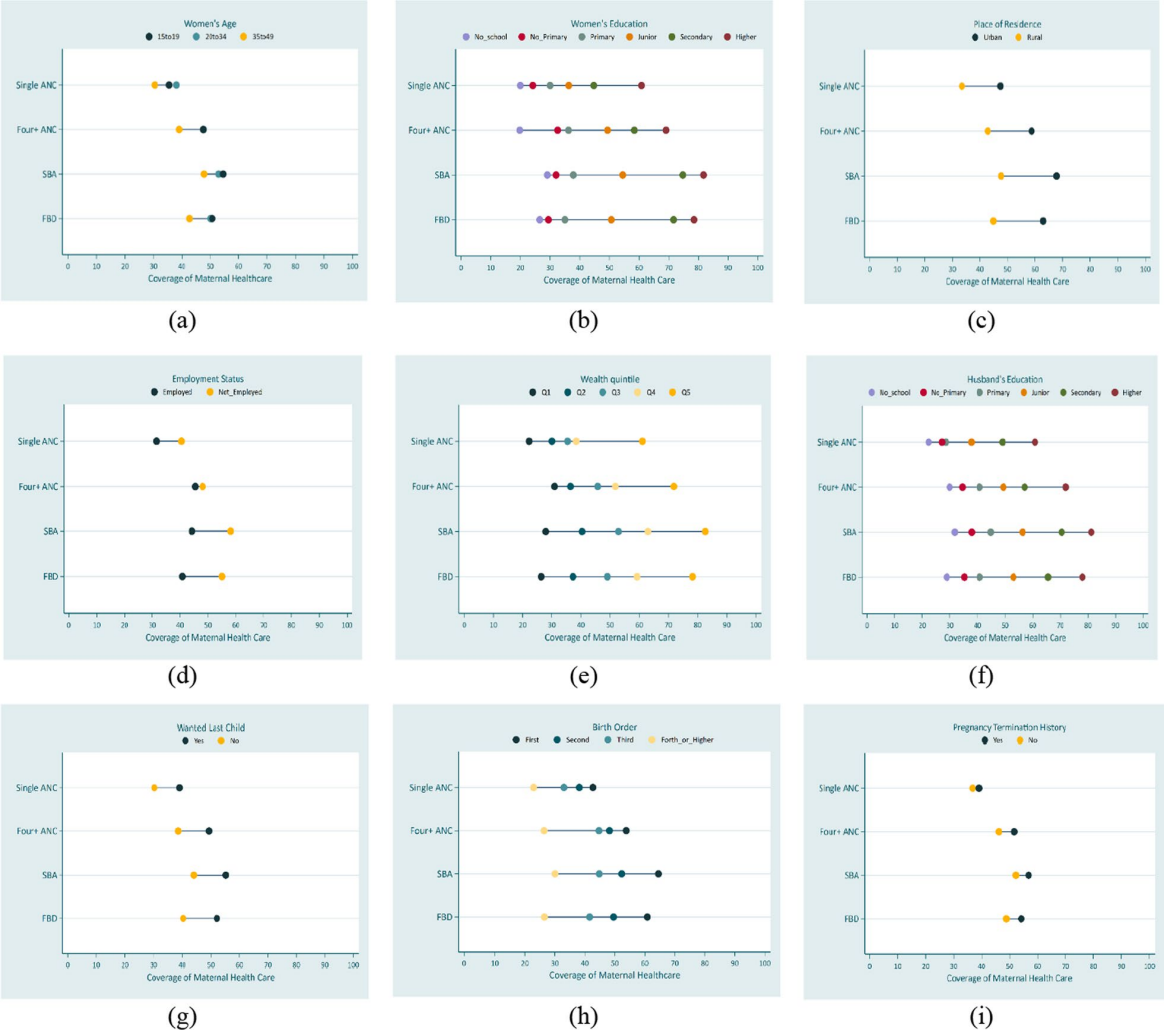
Equiplots of equity strata for maternal healthcare utilization in Bangladesh

**Fig. 5:**
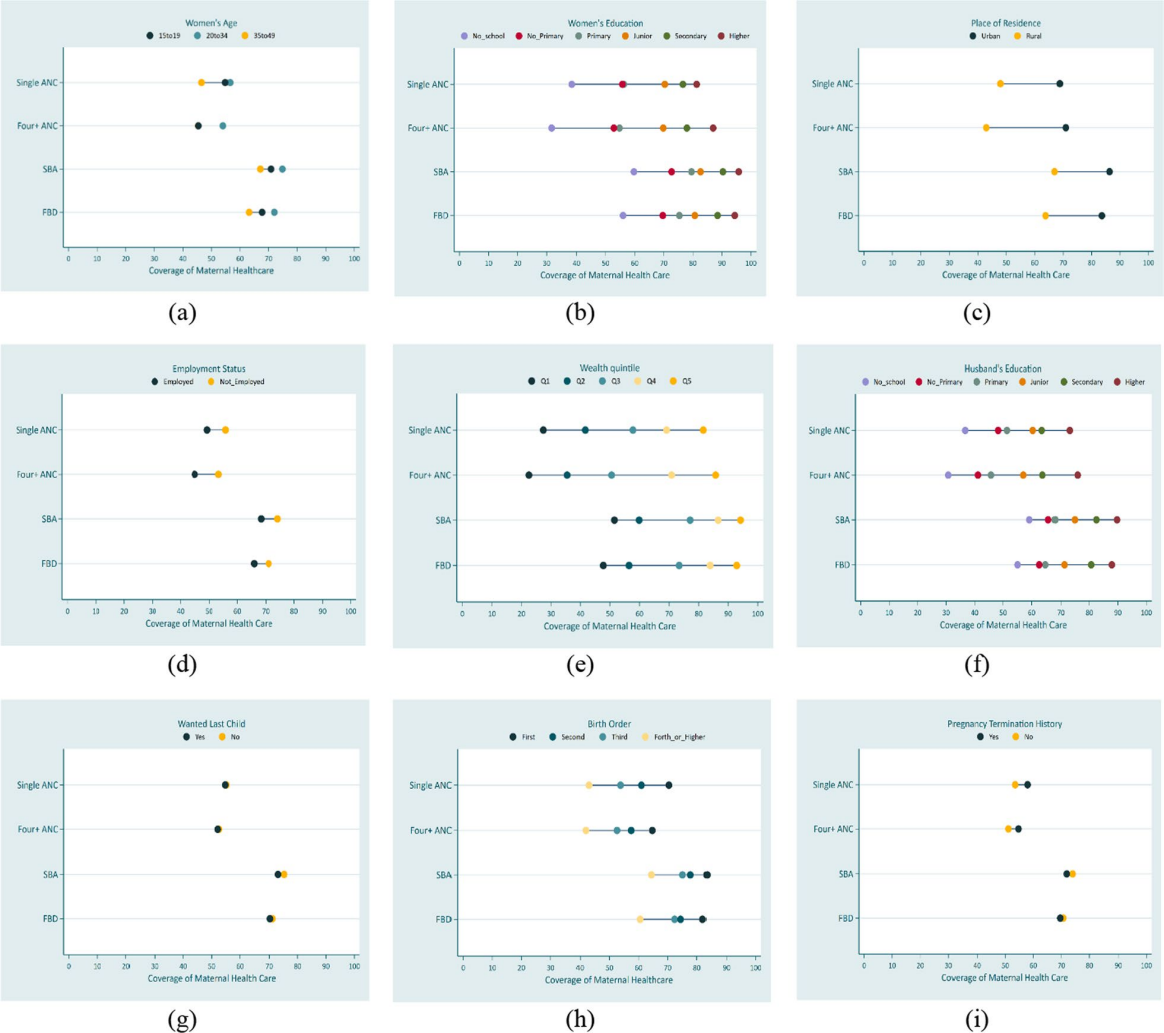
Equiplots of equity strata for maternal healthcare utilization in Pakistan

Figures 4 and 5 show equiplots of maternal healthcare use by common equity strata in Bangladesh and Pakistan. Inequality was present among most of the equity strata for Bangladesh and Pakistan. According to Figs. 4e and 5e of wealth quintile and Figs. 4f and 5f of husband’s education, there was top inequality in all maternal healthcare utilization indicators in Bangladesh and Pakistan. Figures 4h and 5h show the bottom inequality in birth order for all maternal healthcare utilization indicators for both countries.

## Discussion

Our study based on the latest DHS data of Bangladesh and Pakistan (2017–2018) clearly demonstrates inter-country and intra-country inequality in the utilization of maternal healthcare services (single ANC, four + ANC, SBA, and FBD) by common equity strata. The utilization of maternal healthcare services was overall higher in Pakistan than in Bangladesh. However, inequities in maternal healthcare utilization by rich-poor, urban–rural, educational attainments, last birth order, and employment status are prevalent in both countries. In Pakistan, inequities in maternal healthcare utilization by wealth, women's education, and husband’s education were higher to some extent than in Bangladesh. In LMICs of Asia and Africa, greatest inequality was observed in the utilization of maternal healthcare for ANC and SBA services [49].

In Bangladesh, inequality in SBA than other maternal healthcare services by rich-poor, women's education, and husband's education exists in a larger extent. In Pakistan, across different maternal healthcare services, inequality was highest in at least four ANC by skilled providers by wealth, women's education, and their husband's education. This finding is also consistent with the studies in Ethiopia [50], Zambia [51], Mali [52], Nigeria [53], and Ghana [54], which have reported rich-poor and education-based inequality in the utilization of maternal healthcare services. Studies in Bangladesh [55], rural Ethiopia [56], and Myanmar [57] found inequitable distribution in the utilization of SBA; and in India [58] and Pakistan [59], the utilization of at least four ANC by skilled providers was more inequitable. The economic status of women is very crucial in healthcare. Women of the richest wealth quintile have affordability for health services, while the poorest women can hardly afford out-of-pocket payments for any health emergencies [14, 60, 61]. Thus, low economic condition prohibits women from receiving essential maternal healthcare services and instigate rich-poor inequality in society. Educated women and educated husbands exhibit higher health awareness and health-seeking behaviour, and better decision-making ability toward healthcare use than less educated groups [62, 63]. Higher educated women and their husbands are more concerned about modern treatment, more aware of skilled birth attendants, and improved perception of pregnancy complications [64]. Consequently, the differences in perception about the importance and necessity of healthcare among higher educated and non-educated women and their husbands increase the inequality in maternal healthcare.

Inequality was greater for the last birth order favoring the first-order child in receiving medically trained birth attendance in Bangladesh. In Pakistan, inequality was greater for the last birth order favoring the first-order child in visiting a single ANC by the skilled provider. This is also consistent with results of other studies from developing countries, which suggest that women receive higher maternal care for their first child than for subsequent deliveries [58, 62]. Maternal healthcare utilization for the first-order child is higher since women might think their first pregnancy was riskier than subsequent pregnancies [8, 16]. Another possible reason for being less likely to use maternal healthcare for higher-order children might be that mothers with subsequent births become confident and experienced on many aspects of maternal health services, so they have a tendency to believe that they have no further need for some services which they can manage by themselves or at the family level [64]. Therefore, inequality arises with subsequent births.

Results from RR and RD indicate inequality in the utilization of all four maternal healthcare indicators favouring urban and unemployed women. In Bangladesh, the urban–rural inequality was greater in SBA, while it was greater for at least four ANC by the skilled provider in Pakistan. These results also accord with earlier studies, where wide urban–rural disparities for at least four ANC by the skilled provider were observed in Ecuador [48], Rwanda [65], and Nepal [66]. Lack of transportation, long distances to health centers, and bad roads might restrict rural women from accessing to maternal healthcare services [67]. A considerable number of public and private healthcare facilities in urban areas and available transports and improved roads increase healthcare utilization among urban women [64]. Thus, the difference in infrastructure and health facilities prompts urban–rural inequality.

Inequality was greater in FBD, favouring the unemployed women in Bangladesh, while this was true for at least four ANC visits by the skilled provider in Pakistan. Studies conducted in Bangladesh [13], Indonesia [68], and Pakistan [69] have found that working women are less likely to use maternal healthcare services than their non-working counterparts. These findings are inconsistent with studies in Cambodia [34] and Benin [70], where maternal healthcare utilization was greater among working women. Heavy workload during pregnancy and limited time to visit health centres are possible reasons for working women not using maternal health services and rising inequality with employment status [13, 68].

The higher utilization rate of maternal healthcare services in Pakistan compared to Bangladesh is most probably associated with a better health system with a diverse range of private hospitals, private clinics, and other private providers, along with government hospitals and health centres [71]. In addition, re-structuring health policies, initiating vertical programs and introducing Public Private Partnerships (PPP), and improving human resource development and infrastructure by making Basic Health Units and Rural Health Centers might initiate greater utilization of maternal healthcare in Pakistan [72]. Moreover, a continuous assessment of maternal mortality causes and the attempt of government and local organizations of Pakistan to reduce maternal mortality through improving overall maternal healthcare utilization might reduce maternal deaths [15, 73].

The utilization of SBA was highest among the highest level of educated women, women in the richest group, women with higher educated husbands, and first-order children compared to their counterparts. This gap between educated and not/less educated groups justifies the reason for higher inequality in SBA than other maternal healthcare indicators in Bangladesh. The possible reason for this gap may be higher educated women and husband may obtain better health messages, richest women may afford added expenses for SBA and women’s perception of not taking risk for giving birth may motivated them to opt for deliveries assisted by qualified providers [10].

Besides, the utilization of four ANC visits by the skilled provider was highest among the highest level of educated women, women in the richest group, women with higher educated husbands, women in urban areas, and unemployed women compared to their counterparts. This gap between advantaged and disadvantaged groups justifies the reason for higher inequality in four ANC visits by skilled providers than other maternal healthcare indicators in Pakistan. The possible reason for this gap may be a lack of quality health services in rural areas, unaware of pregnancy complications, expenses incurred for travel to health facilities, and engaging in informal wage-jobs that prohibit them from receiving ANC care during their pregnancy [14, 68].

Due to a considerable gap between privileged and underprivileged groups in the utilization of maternal healthcare services, the inequality in maternal healthcare utilization by wealth status, women's education, and husband’s education were higher in Pakistan than in Bangladesh. Accessibility and availability of maternal healthcare and health system interventions may be skewed towards the privileged group, resulting in an increase in maternal healthcare inequality in Pakistan [74].

Although policies like cash transfers, voucher schemes, and removing user fees have already been taken to increase utilization and overcome inequality in maternal health in Bangladesh and Pakistan, treatment-related financial supports to vulnerable groups alone may not solve the inequality problems. To avoid inequitable utilization, the primary goal for policymakers should be to focus on eradicating the disparities resulting from socioeconomic status [75]. District/union council officials might identify eligible and needy families, generate, and maintain village funds, build a community transport system for emergency transport to health care facilities, and implement media and education programs. All these community acts would bolster the government's efforts to improve utilization of maternal healthcare services among the underprivileged in the country. There is evidence that disadvantaged groups benefited from a community-participatory intervention targeted towards the disadvantaged population that typically lacks knowledge about healthcare seeking or the importance of health care [4]. Combining social development programs with equality-oriented health policies could be a better solution to combat this crisis (for example, Maldives incorporated a Master Health plan with social safety net) [7]. It is also instructive to comprehend the diversity of techniques that match a country's political, economic, and cultural contexts.

Our study's strength is that we compared inequality between two historically connected countries, Bangladesh, and Pakistan, using extensive nationally representative surveys of the same period. In addition, we used relative and absolute measures to assess inequality in both ANC and delivery. Regardless of the study's strengths, there are several limitations. Due to the use of a cross-sectional study design, causation assumptions could not be drawn in this investigation. As a result, the findings should be explained with caution. We admit that the data on maternal healthcare usage were self-reported, which may not be free from bias. There is also a risk of recall bias due to including women who had a live birth three years preceding the surveys. This bias could result in overestimating or underestimating the utilization of maternal healthcare services. To reduce this effect, the analysis was conducted on the most recent birth during the three years preceding the survey.

## Conclusion

Inequality pertains to maternal healthcare in Bangladesh and Pakistan. Although the utilization of maternal healthcare is higher in Pakistan, inequality by common equity strata (wealth status, educational attainment, birth order of the last child, place of residence, and employment status) is greater among the underprivileged group in Pakistan than in Bangladesh. In Bangladesh, the SBA is the most inequitable maternal healthcare indicator for the equity strata like wealth, education, and birth order. Among all the maternal healthcare indicators in Pakistan, the four ANC visits by skilled providers is the most inequitable service based on wealth, education, place of residence, and employment status. So, just focusing on improving utilization will not be effective for the overall maternal healthcare achievement; instead, including health equality indicators in global and national monitoring frameworks and combining policies based on country context would be more effective in bridging the gap between the privileged and underprivileged groups.

## Supplementary Information

**Additional file 1:** **Table S1.** Variable’s categorization and leveling. **Table S2. **Binary logistic regression of Maternal Healthcare by Equity Strata for Bangladesh and Pakistan.

## Data Availability

The dataset analyzed during the current study are available in the DHS Program website, https://dhsprogram.com/data/available-datasets.cfm.
