# Supplementary material for: Comparison of inequality in utilization of maternal healthcare services between Bangladesh and Pakistan: evidence from the demographic health survey 2017–2018
Source: Reprod Health. 2023 Mar 13;20:43. doi: 10.1186/s12978-023-01595-y (PMC10009948; doi:10.1186/s12978-023-01595-y)
Supplement: Supplementary file 1 — Additional file 1: Table S1. Variable’s categorization and leveling. Table S2. Binary logistic regression of Maternal Healthcare by Equity Strata for Bangladesh and Pakistan. [file 12978_2023_1595_MOESM1_ESM.docx]

**Table S1: Variable’s categorization and leveling**

| **Outcome Variables** | **Description** | **Categories** |
| --- | --- | --- |
| Single ANC Visit (1st trimester) by the Skilled Provider | Single antenatal care visit in the first trimester by the medically trained provider. In Bangladesh, medically trained/ skilled provider includes qualified doctors, nurses, midwives, or paramedics; family welfare visitors; community skilled birth attendants; and sub-assistant community medical officers. In Pakistan, doctors, nurses, midwives, and lady health visitors are considered medically trained providers, and skilled assistance includes doctors, nurses, midwives, lady health visitors, or community midwives. | Categorized into two levels: No, Yes |
| At least Four ANC Visits by the Skilled Provider | Four or more antenatal care visits by the medically trained provider. |  |
| Skilled Birth Attendance | Birth attended by the skilled provider |  |
| Facility-based Delivery | Delivery in a health facility. In Bangladesh, a medically facilitated place of delivery is a public (e.g., medical college hospital, specialized govt. hospital, district/ upazila hospital, maternal and child welfare centers, uh and family welfare center, and community clinic), private (e.g., private medical college hospital, private hospital, and private clinic) and NGO (e.g., NGO static clinic) facility. In Pakistan, medically facilitated places of delivery include the public sector (e.g., government hospital, rural health center/ mother-child health Centre, basic health unit, and community midwife) and the private sector (e.g., private hospital/clinic). |  |
| **Equity Strata** | **Description** | **Categories** |
| Women’s Age (Years) | Age of the women in years during last live birth | Categorized into three levels: 15–19, 20–34, 35-49 |
| Women’s Education | Women’s level of education | Categorized into six levels: no formal schooling, Primary education not completed, Primary education completed (Grade 5), Junior school completed (Grade 8), Secondary school completed (Grade 10), Higher secondary or above |
| Place of Residence | Place/ Area of Residence | Categorized into two levels: Urban, Rural |
| Household Head | Women are head of their household | Categorized into two levels: Yes, No |
| Household Size | Number of members in the household | Categorized into two levels: 1-5 members, 6 or more members |
| Employment Status | Women’s currently employment/working status | Categorized into two levels: Currently employed, Not currently employed |
| Wealth Quintile | In DHS calculated wealth index (WI) using data from the DHS databases on household asset ownership, household characteristics, household source of drinking water, and household sanitary facilities [26]. The principal components analysis (PCA) technique is used to construct the WI and assigns a score to each household based on chosen household assets. We used the constructed WI in the DHS to divide the population into wealth quintiles. | Categorized into five levels: Poorest, Poorer, Middle, Richer, Richest |
| Husband’s Education | Husband’s level of education | Categorized into six levels: no formal schooling, Primary education not completed, Primary education completed (Grade 5), Junior school completed (Grade 8), Secondary school completed (Grade 10), Higher secondary or above |
| Wanted Last Child | The last live birth was wanted/desired | Categorized into two levels: Yes, No |
| Last Live Birth Order | Order of the last live birth of the child | Categorized into four levels: First, Second, Third, Fourth or Higher |
| Pregnancy Termination History | There was any pregnancy termination incidence | Categorized into two levels: Yes, No |

| **Table S2: Binary logistic regression of Maternal Healthcare by Equity Strata for Bangladesh and Pakistan** | | | | | | | | |
| --- | --- | --- | --- | --- | --- | --- | --- | --- |
| **Equity Strata** | **Odds Ratio (Bangladesh)** | | | | **Odds Ratio (Pakistan)** | | | |
|  | **Single ANC Visit (1^st^ trimester) by Skilled Provider** | **At least Four ANC Visits by Skilled Provider** | **Skilled Birth Attendance** | **Facility-based Delivery** | **Single ANC Visit (1^st^ trimester) by Skilled Provider** | **At least Four ANC Visits by Skilled Provider** | **Skilled Birth Attendance** | **Facility-based Delivery** |
| **Women’s Age** (ref. 15-19) |  |  |  |  |  |  |  |  |
| 20-34 | 1.11 (0.10) | 1.00 (0.08) | 0.94 (0.08) | 0.97 (0.08) | 1.07 (0.18) | 1.41 (0.23)* | 1.22 (0.22) | 1.23 (0.22) |
| 35-49 | 0.79 (0.13) | 0.71 (0.11)* | 0.76 (0.11) | 0.73 (0.11)* | 0.72 (0.13) | 1.00 (0.19) | 0.84 (0.17) | 0.82 (0.16) |
| **Women’s Education** (ref. No formal schooling) |  |  |  |  |  |  |  |  |
| Primary education not completed | 1.28 (0.23) | 1.96 (0.34)* | 1.15 (0.19) | 1.16 (0.19) | 2.03 (0.36)* | 2.41 (0.43)* | 1.80 (0.37)* | 1.80 (0.35)* |
| Primary education completed | 1.72 (0.32)* | 2.30 (0.43)* | 1.48 (0.26)* | 1.49 (0.27)* | 2.07 (0.28)* | 2.61 (0.35)* | 2.62 (0.43)* | 2.41 (0.37)* |
| Junior school completed | 2.28 (0.37)* | 3.95 (0.64)* | 2.92 (0.44)* | 2.84 (0.44)* | 3.81 (0.52)* | 4.98 (0.68)* | 3.21 (0.52)* | 3.28 (0.51)* |
| Secondary school completed | 3.24 (0.68)* | 5.69 (1.19)* | 7.22 (1.50)* | 7.00 (1.45)* | 5.26 (0.81)* | 7.61 (1.15)* | 6.30 (1.30)* | 6.05 (1.16)* |
| Higher secondary or above | 6.22 (1.08)* | 9.06 (1.60)* | 10.90 (1.88)* | 10.12 (1.75)* | 7.00 (1.04)* | 14.39 (2.31)* | 15.31 (3.82)* | 13.29 (2.92)* |
| **Place of Residence** (ref. Rural) |  |  |  |  |  |  |  |  |
| Urban | 1.79 (0.13)* | 1.90 (0.13)* | 2.31 (0.17)* | 2.07 (0.15)* | 2.40 (0.20)* | 3.24 (0.26)* | 3.12 (0.30)* | 2.90 (0.27)* |
| **Household Head** (ref. No) |  |  |  |  |  |  |  |  |
| Yes | 1.13 (0.11) | 0.94 (0.91) | 1.00 (0.10) | 1.01 (0.10) | 0.96 (0.12) | 1.26 (0.16) | 1.08 (0.15) | 1.13 (0.16) |
| **Household Size** (ref. 6 or more members) |  |  |  |  |  |  |  |  |
| 1-5 members | 1.00 (0.07) | 1.07 (0.07) | 1.02 (0.07) | 1.06 (0.07) | 1.02 (0.10) | 1.08 (0.10) | 1.02 (0.11) | 1.05 (0.11) |
| **Employment Status** (ref. Not currently employed) |  |  |  |  |  |  |  |  |
| Currently employed | 0.68 (0.05)* | 0.90 (0.06) | 0.57 (0.04)* | 0.56 (0.04)* | 0.77 (0.09)* | 0.71 (0.08)* | 0.76 (0.09)* | 0.79 (0.10)* |
| **Wealth Quintile** (ref. Poorest) |  |  |  |  |  |  |  |  |
| Poorer | 1.51 (0.17)* | 1.28 (0.13)* | 1.75 (0.18)* | 1.66 (0.17)* | 1.90 (0.24)* | 1.89 (0.24)* | 1.40 (0.16)* | 1.42 (0.16)* |
| Middle | 1.93 (0.22)* | 1.88 (0.19)* | 2.89 (0.30)* | 2.69 (0.29)* | 3.63 (0.45)* | 3.52 (0.44)* | 3.17 (0.40)* | 3.02 (0.37)* |
| Richer | 2.19 (0.24)* | 2.40 (0.24)* | 4.38 (0.46)* | 4.06 (0.43)* | 5.95 (0.77)* | 8.38 (1.10)* | 6.09 (0.91)* | 5.70 (0.80)* |
| Richest | 5.53 (0.61)* | 5.69 (0.61)* | 12.28 (1.48)* | 10.06 (1.17)* | 11.74 (1.73)* | 20.68 (3.24)* | 15.19 (3.10)* | 14.27 (2.69)* |
| **Husband’s Education** (ref. No formal schooling) |  |  |  |  |  |  |  |  |
| Primary education not completed | 1.30 (0.17)* | 1.24 (0.15) | 1.31 (0.16)* | 1.34 (0.16)* | 1.61 (0.30)* | 1.58 (0.30)* | 1.33 (0.30) | 1.37 (0.26) |
| Primary education completed | 1.39 (0.19)* | 1.61 (0.20)* | 1.75 (0.22)* | 1.69 (0.21)* | 1.82 (0.25)* | 1.90 (0.27)* | 1.47 (0.22)* | 1.50 (0.22)* |
| Junior school completed | 2.12 (0.25)* | 2.29 (0.25)* | 2.77 (0.30)* | 2.77 (0.31)* | 2.63 (0.32)* | 3.00 (0.37)* | 2.06 (0.27)* | 2.02 (0.26)* |
| Secondary school completed | 3.35 (0.57)* | 3.12 (0.52)* | 5.14 (0.89)* | 4.68 (0.79)* | 3.01 (0.35)* | 3.97 (0.47)* | 3.24 (0.43)* | 3.40 (0.43)* |
| Higher secondary or above | 5.39 (0.68)* | 6.02 (0.74)* | 9.29 (1.23)* | 8.71 (1.13)* | 4.69 (0.55)* | 7.10 (0.84)* | 6.00 (0.86)* | 5.91 (0.80)* |
| **Wanted Last Child** (ref. No) |  |  |  |  |  |  |  |  |
| Yes | 1.47 (0.12)* | 1.55 (0.12)* | 1.57 (0.12)* | 1.61 (0.13)* | 0.98 (0.11) | 0.98 (0.11) | 0.89 (0.12) | 0.96 (0.12) |
| **Last Live Birth Order** (ref. Fourth or Higher) |  |  |  |  |  |  |  |  |
| First | 2.51 (0.30)* | 3.24 (0.37)* | 4.24 (0.48)* | 4.30 (0.50)* | 3.16 (0.33)* | 2.55 (0.27)* | 2.77 (0.34)* | 2.96 (0.35)* |
| Second | 2.08 (0.25)* | 2.59 (0.30)* | 2.55 (0.29)* | 2.72 (0.32)* | 2.07 (0.21)* | 1.87 (0.19)* | 1.93 (0.23)* | 1.90 (0.21)* |
| Third | 1.66 (0.23)* | 2.25 (0.29)* | 1.89 (0.24)* | 1.97 (0.25)* | 1.55 (0.17)* | 1.54 (0.17)* | 1.66 (0.20)* | 1.71 (0.20)* |
| **Pregnancy Termination History** (ref. No) |  |  |  |  |  |  |  |  |
| Yes | 1.10 (0.10) | 1.25 (0.11)* | 1.20 (0.10)* | 1.24 (0.11)* | 1.20 (0.10)* | 1.16 (0.10) | 0.90 (0.09) | 0.95 (0.09) |
| Notes: * if p<0.05. Standard error is in parenthesis. | | | | | | | | |
